# The Influence of New Media Literacy on Brand Engagement: Mediating Effects of Perceived Interactivity and Openness and the Moderating Effect of Age

**DOI:** 10.3390/bs15040458

**Published:** 2025-04-02

**Authors:** Changi Song, Eunho Kim

**Affiliations:** 1 Department of Psychology, Jeonbuk National University, Jeonju-si 54896, Jeollabuk-do, Republic of Korea; 2 Department of Management Information Systems, Dong-A University, Busan 49236, Gyeongsangnam-do, Republic of Korea

**Keywords:** new media literacy, perceived interactivity, perceived openness, brand engagement

## Abstract

The shift toward new media has brought changes to market participants. New media literacy has emerged as a necessary competency for consumers, and brand engagement has become a key goal of brand communication. However, despite the established importance of new media literacy, how it affects perceptions and reactions toward brand communication has not been investigated. This study examined how functional and critical consuming literacy, which are components of new media literacy, influence brand engagement through the mediating roles of perceived interactivity and openness and the moderating effect of age. A cross-sectional study was conducted with 260 South Korean adults and a hypothetical brand’s social media post. The results showed that the effect of functional consuming literacy on engagement varied by age: functional consuming literacy in the younger group decreased perceived interactivity, perceived openness, and brand engagement. In the older group, functional consuming literacy preliminarily increased perceived interactivity. Critical consuming literacy enhanced perceived interactivity and openness, positively affecting brand engagement across all ages. This study reframes new media literacy as critical in shaping consumer behavior and brand interactions. These findings suggest that new media literacy is a critical variable in understanding consumer behavior in the new media era.

## 1. Introduction

The rapid advancement of information and communication technology has fundamentally reshaped the landscape of information exchange. Traditionally, mass media such as newspapers and television were characterized by a unidirectional flow of information, where a limited number of professionals controlled the production and dissemination of content. In stark contrast, the convergence of ICT with media has given rise to new media like social media and websites and ushered in an era of “democratization of content production and dissemination” (Dhiman, 2023, p. 1). This paradigm shift is largely attributed to the interactive and accessible nature of new media, which empower individuals to effortlessly access, create, and share content, thereby fostering more efficient and dynamic information sharing.

However, this transformation of the media environment has presented consumers with new challenges. First, the shift towards new media has fundamentally altered the way information is exchanged. For instance, as new media rely heavily on internet networks and digital signals, consumers can now access information only by using internet-connected devices, a departure from traditional media delivery. Furthermore, consumers are exposed to a variety of new types of information that they have not encountered before, such as short-form videos and social media posts. Simultaneously, and paradoxically, the abundance of information within new media hinders effective information seeking itself. The new media environment is saturated with information disseminated by a multitude of users, leading to information overload, which makes it difficult for consumers to identify essential information and increases the likelihood of encountering unrefined information, misinformation, fake news, and propaganda (Dhiman, 2023; Gaozhao, 2021; Yum & Jeong, 2019). To effectively navigate these challenges, consumers need new abilities to access new media information, appropriately interpret the diverse types of information, and protect themselves from information overload and misinformation. This necessitates the development of new media literacy, which encompasses skills such as information navigation and critical thinking about content within new media (Celik et al., 2021; Tugtekin & Koc, 2020).

The impact of the shifting media landscape is not isolated to consumers. As new media grow, brands have increasingly adopted them to communicate with consumers (Gao & Feng, 2016; Khanom, 2023; Voorveld, 2019). Notably, social media platforms like Facebook and Instagram have emerged as the primary channels, as they facilitate direct interaction between brands and consumers (Arya et al., 2022; Mangold & Faulds, 2009; Scarpi, 2010; Voorveld, 2019). However, the utilization of social media has also brought about changes in brand communication. In social media communication, consumers are no longer passive recipients of information, but actively participate in brand value creation by expressing opinions and sharing brand experiences (Arya et al., 2022; Prahalad & Ramaswamy, 2004; Ramaswamy & Ozcan, 2016; Zadeh et al., 2019). Therefore, social media brand communication now emphasizes fostering active and dynamic brand–consumer relationships (Brodie et al., 2011; Kulikovskaja et al., 2023). While academia and practitioners have sought to explain and respond to these changes in brand communication, traditional communication concepts such as involvement or participation have been criticized for their limitations in reflecting the dynamic interactions between brands and consumers (H. J. Kim et al., 2019). Consequently, there has been a growing interest in a new concept that captures these characteristics: brand engagement (Brodie et al., 2011; Hollebeek et al., 2014; H. J. Kim et al., 2019; Kulikovskaja et al., 2023; Lim & Rasul, 2022).

Despite new media literacy and brand engagement gaining attention in similar technological and social contexts, research exploring their relationship remains limited. While prior research on new media literacy has offered diverse academic perspectives (Arsenijević & Andevski, 2016; Chen et al., 2011; Gogus et al., 2024; Koc & Barut, 2016; Lin et al., 2013; Luo et al., 2022; Orhan, 2023; Shahzad & Khan, 2024; Veeriah, 2021; Xiao et al., 2021; Yum & Jeong, 2019), it often regards it as an adaptive competency for navigating the digital landscape, such as a means to address misinformation (Shahzad & Khan, 2024; Yum & Jeong, 2019). Consequently, there is a lack of research on how new media literacy influences consumer behavior in brand communication contexts. However, given that new media literacy involves information acquisition and processing through new media and that new media platforms are increasingly used for brand communication, it is likely that new media literacy influences consumers’ reactions toward contemporary brand communication, including brand engagement.

To address this research gap, this study examined the relationship between new media literacy and brand engagement within social media. This study had two primary objectives. First, we aimed to investigate the influence of functional and critical consuming literacy, two key components of new media literacy, on brand engagement. These components are especially relevant to how consumers perceive and assess information on new media and thus are expected to be closely related to consumer responses to brand communication on social media. Second, we also aimed to examine the mediating roles of perceived interactivity and openness of brands in the relationship between new media literacy and brand engagement. These perceptions are likely influenced by new media literacy, as consumers form them based on brand behaviors and messages on social media. Furthermore, previous research has identified these concepts as antecedents of brand engagement (Labrecque, 2014).

Additionally, this study explored the role of age as a moderating variable affecting the relationship between new media literacy and brand engagement. While concerns about the digital divide based on generation have been raised, age has often been regarded as a predictor of new media literacy (Choi et al., 2018; Gogus et al., 2024; Shim, 2022). However, new media usage has grown between age groups, including older adults (Cotten et al., 2022; Sinclair & Grieve, 2017). Therefore, rather than simply viewing the relationship between age and new media literacy as linear, examining whether individuals with similar levels of new media literacy exhibit differences in how they process new media information based on age can contribute to a more nuanced understanding of the relationship between age and new media literacy.

This study offers a novel perspective by examining new media literacy as a consumer characteristic that influences brand–consumer interactions, moving beyond its traditional role as an adaptive factor. This approach expands our understanding of contemporary consumer behavior by considering new media literacy a key variable relevant to today’s new media-based branding and marketing. Building on this, we propose that in the current media landscape, where new media are a primary communication channel for brands, new media literacy should be considered a key factor influencing consumer responses and behaviors. This contributes to both theory and practice by providing a more comprehensive framework for understanding consumer behavior in the digital age and offering valuable guidance for developing effective marketing communication strategies tailored to consumers’ new media literacy levels.

Following this introduction, we provide a literature review and develop hypotheses on the relationships among new media literacy, brand engagement, and perceived interactivity and openness. Additionally, we examine existing research and formulate research questions regarding the relationship between age and new media literacy. We then present our empirical study, which tests these hypotheses and research questions. Finally, we discuss our key findings and offer suggestions for future research.

## 2. Literature Review and Hypothesis Development

### 2.1. New Media Literacy

As media-related technologies have advanced, literacy has expanded to encompass competencies beyond basic reading and writing skills (Xiao et al., 2021). In the contemporary media landscape, literacy needs to encompass the ability to critically evaluate and produce media content, as well as to access and understand such content (Chen et al., 2011; Koltay, 2011; Lin et al., 2013). Accordingly, Chen et al. (2011) introduced a framework for new media literacy that categorizes competencies along two axes: the consumption–production continuum and the functional–critical continuum. Based on those axes, this framework identifies four core components of new media literacy. First, functional consuming literacy refers to the ability to access new media content and comprehend the textual meanings of content. This includes technical skills for operating the hardware or software for consuming content, as well as the ability to interpret textual meanings within content (Lin et al., 2013). Second, critical consuming literacy involves the ability to critique new media content, construe the embedded social, economic, and political meanings, and understand the creation processes and purposes behind new media content. Key skills include recognizing the individual components of a message (e.g., author, format, audience), integrating and reconstructing messages from one’s perspective, questioning the credibility of media content, and engaging in critical analysis (Lin et al., 2013). Third, functional prosuming literacy pertains to the ability to create and distribute new media content. It encompasses technical proficiency in operating devices to produce and distribute new media content, as well as the capacity to copy or remix content to create new ones (Lin et al., 2013). Finally, critical prosuming literacy extends beyond the mere production of content, focusing on expressing one’s beliefs and values, negotiating with others in the new media space, and considering the potential impacts of these actions. This requires social skills that enable interactive and critical communication and the ability to inject one’s values and ideologies into the content (Lin et al., 2013; Tugtekin & Koc, 2020).

As the importance of new media literacy continues to grow, a substantial body of research has explored this construct (Arsenijević & Andevski, 2016; Chen et al., 2011; Gogus et al., 2024; Koc & Barut, 2016; Lin et al., 2013; Luo et al., 2022; Orhan, 2023; Shahzad & Khan, 2024; Veeriah, 2021; Xiao et al., 2021; Yum & Jeong, 2019). While these studies have offered diverse academic perspectives, they often focus on the adaptive nature of new media literacy, highlighting its importance in navigating the digital landscape. However, this emphasis on adaptability overlooks the broader implications of new media literacy in today’s society. New media literacy is linked to acquiring and processing information. As new media platforms increasingly dominate as the primary medium for information exchange, including brand–consumer communication, their influence transcends mere adaptability, carrying profound implications for individual lives, including shaping how consumers evaluate and interpret brand-related information. Consequently, this competency can influence consumer cognition, affect, and behavior toward brands. Therefore, new media literacy should be recognized as a critical construct shaping consumer attitudes, beliefs, and behaviors in the new media era.

Recently, Polizzi (2025) investigated how functional and critical literacy facilitate social participation. The study found that experts and advocates strategically employ both functional and critical digital literacies to engage in a range of activities, such as consuming news, sharing aspects of public life, participating in political discourse, and organizing campaigns. While this research highlights the impact of new media literacy on individuals’ direct behaviors, it falls short of adequately explaining the mechanisms through which functional and critical literacy shape consumer behavior in the context of new media engagement. To address this gap, the present study examined how consumers’ functional and critical consuming literacy influence their perceptions of brand activities on social media, and how these perceptions in turn affect consumer behavior, particularly brand engagement. By clarifying the distinct effects of functional and critical consuming literacy on consumer perceptions, this study aims to provide strategic insights into how these dimensions of new media literacy can be leveraged in brand communication within the new media landscape.

### 2.2. Brand Engagement on Social Media

The proliferation of new media has transformed the interaction between brands and consumers into a more dynamic and bidirectional process (Baumöl et al., 2016). Particularly on social media, which facilitates interaction among users, consumers are now actively participating in building relationships with brands (Arya et al., 2022; Hennig-Thurau et al., 2010; Prahalad & Ramaswamy, 2004; Ramaswamy & Ozcan, 2016; Zadeh et al., 2019). As social media has become a primary context for the development of brand–consumer relationships, both academia and industry have recognized the need for new perspectives that focus on the interactive aspects of these relationships. In line with this, brand engagement has become a focal point of interest.

Brand engagement is defined as the behavioral manifestation of consumers motivated by a specific brand–consumer relationship that extends beyond mere purchase behavior (van Doorn et al., 2010). These behaviors can be manifested online and offline, such as word-of-mouth communication, recommendations, assisting other consumers with purchases, blogging, and writing reviews (Brodie et al., 2013; Sashi, 2012). On social media, consumer engagement can be measured through overt interactive behaviors such as comments, retweets, shares, and likes (Barger et al., 2016; Jeseo et al., 2024; Schivinski et al., 2016).

Unlike related marketing constructs like brand involvement, brand image, brand satisfaction, and brand experience, brand engagement emphasizes the dynamic and interactive nature of the brand–consumer relationship (Brodie et al., 2011; L. Hollebeek, 2011; L. D. Hollebeek et al., 2014). Moreover, brand engagement plays a central role in the relationships between these constructs (Brodie et al., 2011). For instance, brand engagement mediates the effects of brand involvement on self-brand connection and brand usage intentions (L. D. Hollebeek et al., 2014). Today, brand engagement is a central element of brand communication and a key metric for assessing brand performance (Kaplan & Haenlein, 2010; Lemon & Verhoef, 2016).

### 2.3. Antecedents of Brand Engagement: Perceived Interactivity and Openness

Previous research has demonstrated that consumers’ experiences with factors such as flexibility (Gligor & Bozkurt, 2021), relative perspective (Nair & Little, 2016), and empathy (Dessart & Pitardi, 2019), as well as their abilities to evaluate, analyze, interpret, and connect (Holt, 2016; Holt & Cameron, 2010; Li et al., 2020), significantly influence the development of brand engagement in the context of online marketing activities.

Because brand engagement stems from the interaction between consumers and brands, factors that foster positive brand–consumer relationships can also enhance brand engagement (Labrecque, 2014; Zhong et al., 2021). On social media, the relationship between consumers and brands is often characterized as parasocial, because although it differs from actual interpersonal relationships, it is perceived by consumers as similar to relationships between people (E.-H. Kim et al., 2019; H. J. Kim et al., 2019; Labrecque, 2014). Labrecque (2014) suggested that parasocial relationships are facilitated by two factors—interactivity and openness—that contribute to fostering intimacy in interpersonal relationships.

Perceived interactivity refers to the extent to which consumers perceive their interactions with a brand as similar to interpersonal communication, fostering a sense of social presence (Thorson & Rodgers, 2006). Several researchers have suggested requirements for perceiving brand interactivity. According to Labrecque (2014), perceived interactivity is high when consumers believe the brand actively listens and responds to their messages. Vendemia (2017) suggests two specific cues for perceiving brand interactivity: a brand’s direct responses to consumer messages (e.g., replying to comments) and tailored responses that address specific consumer needs (e.g., providing relevant content). Also, consumers can evaluate a brand’s interactivity not only from brand’s reaction to them but also through social learning, that is, consumers can also evaluate a brand’s interactivity through social learning, that is, by observing how a brand interacts with other consumers (Vendemia, 2017).

Furthermore, some researchers have focused on the technical aspects of brand–consumer interaction in addition to the qualitative aspects (e.g., inferences about brand characteristics). For example, France et al. (2016) posit that perceptions of interactivity are influenced by both an overall evaluation of whether the brand seems willing to listen to consumers and perceptions of the characteristics of the online interactions provided by the brand, such as the provision of efficient and convenient means. More recently, Cheung et al. (2020) synthesized previous research and identified five sub-dimensions of interactivity: entertainment interactivity (Is the interaction perceived as fun and playful?), customization interactivity (Is the interaction customized for the specific consumer?), interactivity ease of use (Can consumers interact brand easily?), cognitive information–transfer interaction (Is interaction with other users possible?), and cognitive up-to-date information interactivity (Does consumer interaction provide up-to-date information?).

Openness, as defined by Labrecque (2014), refers to the perceived degree of self-disclosure by the brand. In interpersonal relationships, self-disclosure involves sharing private information (Reis & Shaver, 1988). However, in consumer–brand interaction, the information shared should go beyond product attributes, operational statistics, or other generic data. Instead, it involves providing new information about the process behind the brand’s products or advertisement creation (Huaman-Ramirez et al., 2022). For instance, behind-the-scenes content showcasing the development of products or advertisements is considered brand self-disclosure, enhancing perceptions of the brand’s openness (Moon, 2015).

Openness is also linked to consumers’ perception of receiving sufficient information from the brand—transparency (Eggert & Helm, 2003; Labrecque, 2014). Prior research has identified several sub-dimensions of transparency, highlighting the key factors that contribute to consumers’ perceptions of a brand providing adequate information (Montecchi et al., 2024; Sansome et al., 2024; Schnackenberg et al., 2021). Unsurprisingly, the availability of information, which reflects consumers’ perceptions of the extent to which a brand discloses its information, is considered a fundamental condition. However, Sansome et al. (2024) assert that information availability is one of the requirements of transparency perceptions, and the attributes of the information and the brand must also be considered. Specifically, three crucial elements can be identified. First, the objectivity of information refers to consumers’ perception of the accuracy and factual basis of the information disclosed by the brand. Second, the comprehensibility of information entails the extent to which consumers can clearly and easily understand the information provided by the brand. Finally, the intentions of information pertain to consumers’ belief that the brand’s disclosure of information stems from genuine motives rather than external pressures or ulterior motives.

### 2.4. Hypothesis Development

This study examined the relationships between the key variables based on their definition and prior theoretical frameworks. As mentioned in the Introduction, this study focused on functional and critical consuming literacy, because these components are especially relevant to processing new media information and thus are expected to affect consumer perceptions of brand communication on social media. We predicted that functional and critical consuming literacy impact perceived interactivity, openness perceptions, and ultimately brand engagement. Specific predictions and rationale for each relationship and the entire mediation model are given below.

As mentioned, functional consuming literacy includes the two types of ability. One is the competency to utilize new media hardware and software to access new media contents and information, and the other is the competency to comprehend textual meanings of various forms of new media content (Chen et al., 2011; Lin et al., 2013). Technical facilitation and ease of use in brand–consumer communication enhance the consumers’ perceptions of interactivity (Cheung et al., 2020; France et al., 2016). Prior research mainly regards these factors as brands’ or communication means’ features, which can be achieved when a consumer is already familiar with the specific communication means. Consumers with high functional consuming literacy are skilled in acquiring information through various new media platforms, and thus are technically prepared for new media interaction with brands. This technical characteristic of functionally literate consumers may facilitate perceptions of brand interactivity. Based on this rationale, we set the research hypothesis about the relationship between functional consuming literacy and perceived interactivity as:

**H1.**  *Functional consuming literacy will have a positive effect on the perceived interactivity with the brand.*

Another facet of functional consuming literacy pertains to the interpretation of literal meanings within new media content (Chen et al., 2011; Lin et al., 2013). Prior studies have indicated that brands perceived as providing easily understandable information foster consumer perceptions of transparency and openness (Montecchi et al., 2024; Sansome et al., 2024; Schnackenberg et al., 2021). However, this phenomenon can be attributed not only to the brand’s communication attributes but also to consumer characteristics. Specifically, consumers possessing a capacity to comprehend brand communications are more likely to perceive the provided information as readily understandable. Consumers with high functional consuming literacy are adept at deciphering information disseminated by other users on new media platforms. Consequently, they are expected to effortlessly extract the literal meanings of brand-generated information that adheres to new media conventions. Thus, functional consuming literacy may positively influence consumer perceptions of brand openness. Accordingly, the following research hypothesis was proposed:

**H2.**  *Functional consuming literacy will have a positive effect on the perceived openness of the brand.*

We predicted the effect of critical consuming literacy drawing on the persuasion knowledge model (Friestad & Wright, 1994). According to this model, consumers are not passive recipients of a brand’s persuasion attempts (e.g., advertising and communication). Instead, they actively react to persuasion using relevant knowledge, namely persuasion knowledge. This cognitive structure comprises beliefs about marketers’ persuasion tactics, the psychological mechanisms underlying those tactics, their effectiveness or appropriateness, and consumers’ own tactics for coping with them.

Rahmani (2023) proposes a model of the psychological process of persuasion knowledge acquisition, activation, and reaction. This model suggests that the specific persuasion knowledge activated influences the direction of the response toward persuasion attempts. If consumers recognize a brand’s ulterior motive underlying persuasion, negatively valenced persuasion knowledge is activated, leading consumers to perceive the brand as having manipulative intent (Rahmani, 2023; see also Wentzel et al., 2010). Thus, factors that increase the accessibility of a brand’s ulterior motive lead consumers to attribute the persuasion to negative intent and ultimately to react defensively.

Consumers with high critical consuming literacy are adept at recognizing the author, creation process, and underlying intent of new media content (Chen et al., 2011; Lin et al., 2013). Consequently, when exposed to a brand’s new media communication (e.g., a social media post), they are less likely to view it as typical user-generated content. Instead, they tend to scrutinize the reasons behind the message and are more prone to discern the commercial intent of brand communication. This heightened awareness can trigger negatively valenced persuasion knowledge, leading these consumers to evaluate the brand communication’s motive unfavorably. However, displaying genuine desire for communication is one of the key antecedents of perceived interactivity (France et al., 2016). Consumers’ suspicion of ulterior motives underlying information disclosure harms perceived openness (Sansome et al., 2024; Schnackenberg et al., 2021). In other words, perceptions of a brand’s interactivity and openness are contingent upon consumers’ belief that the brand is communicating with sincerity and without ulterior motives. Therefore, it was predicted that consumers’ critical consuming literacy dampens perceived interactivity and openness. Based on this reasoning, we proposed the following research hypotheses:

**H3.**  *Critical consuming literacy will have a negative effect on perceived interactivity with the brand.*

**H4.**  *Critical consuming literacy will have a negative effect on the perceived openness of the brand.*

Lastly, Labrecque (2014) posits that perceived interactivity and openness foster brand engagement by cultivating positive brand–consumer parasocial interactions. If functional and critical consuming literacy are associated with perceived interactivity and openness, it is anticipated that these literacies will also exert influences on brand engagement. Building upon this, we proposed the following research hypotheses, which predict the indirect effects of new media literacy:

**H5.**  *Functional consuming literacy will positively influence engagement, mediated by perceived interactivity and perceived openness.*

**H6.**  *Critical consuming literacy will negatively influence engagement, mediated by perceived interactivity and perceived openness.*

### 2.5. The Effect of New Media Literacy Between Age Groups

Studies examining the relationship between new media literacy and demographic characteristics consistently report that age consistently predicts new media literacy (Choi et al., 2018; Gogus et al., 2024; Shim, 2022). These studies have particularly observed that older adults consistently demonstrate lower levels of new media literacy relative to younger groups, providing empirical support for the digital divide across generations. However, prior research on the relationship between age and new media literacy has predominantly focused on their linear relationship.

Nonetheless, the effect of new media literacy can vary across ages. Indeed, some studies have empirically demonstrated this possibility. Choi and Jeong (2019) found that functional consuming literacy exerts a negative impact on online privacy protection behaviors, with this effect being more pronounced among older adults. The researchers interpreted this finding as resulting from the lack of institutional education on privacy protection for older individuals, which may mitigate the negative effects of functional consuming literacy. Similarly, K.-H. Kim and Yoo (2020) found that the ability to utilize new media devices affects different aspects of psychological well-being depending on age. Specifically, new media device proficiency was found to enhance self-efficacy in middle-aged adults while improving life satisfaction in older adults. The researchers explained this difference by noting that for middle-aged individuals, new media device proficiency is related to work performance, whereas for retired older adults, it is associated with daily life. These studies suggest that age-related differences in attitudes toward and patterns of new media use may moderate the magnitude or direction of the effects of new media literacy.

Moreover, motivational differences associated with age may influence how individuals process social information. According to socioemotional selectivity theory, different goals are activated depending on the perceived remaining lifespan (Carstensen, 2006). When individuals perceive a long future ahead, they are more likely to focus on acquiring new information to prepare for the future. In contrast, when individuals perceive a limited remaining lifespan, they prioritize regulating their emotional state to enhance psychological well-being. These motivational differences also affect how social information is processed. Younger individuals with a perceived longer future tend to pay greater attention to negative information and remember it better. Conversely, older individuals with a shorter perceived future pay more attention to positive information, process it more deeply, remember it better, and evaluate their environment more positively (Carstensen et al., 2003; Carstensen & Turk-Charles, 1994; Charles et al., 2003; Mather & Carstensen, 2003).

These motivational differences associated with age and their impact on information processing may interact with the ability to process new media messages, thereby influencing how individuals evaluate brand messages on social media. In other words, even among individuals with similar levels of new media literacy, age may lead to divergent evaluations of brand messages. Consequently, the effects of new media literacy are likely to vary across consumer age groups.

Accordingly, this study aimed to examine whether the effects of new media literacy differ across consumer age groups by addressing the following research questions:

RQ1: Does the effect of functional consuming literacy on perceived interactivity vary by age group?
RQ2: Does the effect of functional consuming literacy on perceived openness vary by age group?
RQ3: Does the effect of critical consuming literacy on perceived interactivity vary by age group?
RQ4: Does the effect of critical consuming literacy on perceived openness vary by age group?
RQ5: Does the indirect effect of functional consuming literacy on brand engagement, mediated by perceived interactivity and openness, vary by age group?
RQ6: Does the indirect effect of critical consuming literacy on brand engagement, mediated by perceived interactivity and openness, vary by age group?

**Figure 1:**
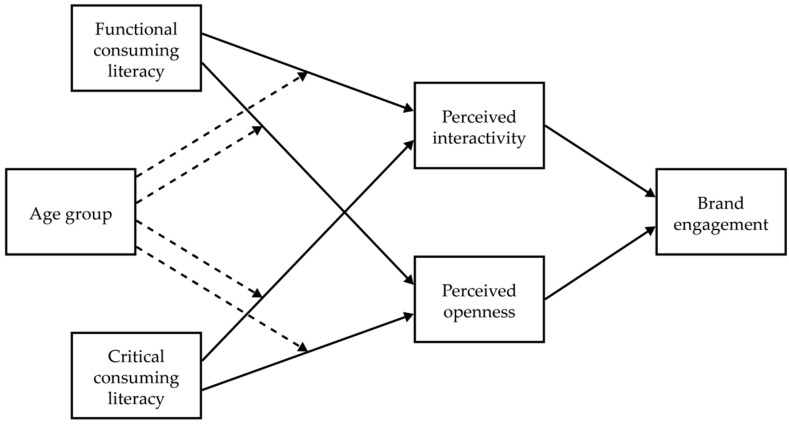
Conceptual model. Dotted lines indicate research hypotheses.

The conceptual model of this study, illustrating the hypothesized relationships and research questions, is presented in Figure 1.

## 3. Materials and Methods

### 3.1. Overview

We employed a cross-sectional research design to investigate the effects of functional and critical consuming literacy on perceived interactivity, perceived openness, and brand engagement, and the moderation by age group, within the context of new media brand communication. We chose social media, one of the new media representatives (Castells, 2011), especially Facebook and Instagram, the most widely used social media platforms in South Korea (Y. Kim, 2024), as our research context. Participants were exposed to a hypothetical hotel brand’s post and then evaluated the hotel brand. This approach, frequently employed in studies examining the influence of brand or consumer characteristics on perceptions or reactions toward a brand (e.g., Carnevale et al., 2018; Huang et al., 2022; Jiao et al., 2022; Penttinen, 2023), was adopted to simulate a realistic social media experience and to minimize the potential impact of participants’ pre-existing brand knowledge or experiences (Carnevale et al., 2018).

### 3.2. Participants and Procedure

Participants were recruited through a professional South Korean survey company. The company sent an email invitation to potential participants that directed them to an online survey platform. Potential participants responded to screening questions regarding their gender, age, and social media platform usage. Participants who were not within the focal age groups (20 to 39 or 60 and above) or had not used Facebook or Instagram were excluded. Those who had used both platforms were further asked to indicate which platform they were more familiar with. A total of 260 participants were recruited through this process, divided equally into two age groups: 130 younger adults aged 20 to 39 (mean age = 32.2 years, 65 women) and 130 older adults aged 60 and above (mean age = 63.9 years, 65 women).

After the screening process, participants completed questionnaires measuring functional and critical consuming literacy. Subsequently, they viewed a hypothetical hotel brand’s post, which was tailored to the social media platform they had used or were more familiar with, to ensure the stimuli were relevant to their typical online experience. Participants then evaluated the hotel brand’s interactivity, openness, and brand engagement. After the evaluation, participants were thanked, and the study concluded.

### 3.3. Materials

The stimulus material for this study, a hypothetical hotel brand’s post, was developed based on established research examining brand interactivity and perceived openness in social media contexts (Labrecque, 2014; Vendemia, 2017). The post featured an image of artwork within the hotel, accompanied by a brief description providing back-story about the artwork, mirroring typical brand content on social media. The post also included three user comments and a response from the brand, simulating an interactive communication exchange (Appendix A).

Given that brand communication behavior can significantly impact perceptions of interactivity and openness (Labrecque, 2014; Vendemia, 2017), the stimulus was designed to represent neutral levels of these factors. This approach aimed to ensure sufficient variance in perceived interactivity and openness, preventing ceiling or floor effects, and to minimize the confounding effect of highly salient interactivity or openness cues in the stimulus.

A pilot study was conducted with 60 adults (not participating in the main study) to validate the neutrality of the stimulus. Participants were asked to rate the stimulus on a 7-point scale for perceived interactivity and perceived openness. The results confirmed that the stimulus was perceived at the midpoint of the scales (4) for both variables: interactivity, *M* = 4.00, *SD* = 1.51, *t*(59) = 0.00, *p* = 1.000; openness, *M* = 4.03, *SD* = 1.30, *t*(59) = 0.20, *p* = 0.843.

### 3.4. Measurements

All the variables below were measured using 7-point Likert scales. A higher score indicated agreement with the item’s statements or evaluating the brand positively.

Functional and critical consuming literacy of new media literacy were measured by Choi et al. (2018)’s scale, which is modified from Koc and Barut (2016). The original measurement covers all four components of Chen et al. (2011), but we only used 13 items for functional consuming literacy (e.g., “I can use internet messaging programs”) and 7 items for critical consuming literacy (e.g., “I can distinguish different functions of media (communication, entertainment, etc.)”). Cronbach’s alphas of both scales were 0.93 and 0.95, respectively.

Perceived interactivity and openness were measured by Kim and colleagues’ translated scale (E.-H. Kim et al., 2019) of Labrecque (2014). Perceived interactivity was measured by four items (e.g., “[Brand] will talk back to me if I post a message”), while perceived openness was measured by three items (e.g., “[Brand] is open in sharing information”). Cronbach’s alphas were 0.89 and 0.84, respectively.

Brand engagement was measured as visible and measurable actions of consumers toward brand content on social media, which was based on the operational definition of Barger et al. (2016). Specifically, four items modified from Jang et al. (2023) and Triantafillidou and Siomkos (2018) were used. The items were: “I intend to click like to the brand’s post,” “I intend to leave a comment on the brand’s post,” “I intend to visit the brand’s page and read other posts,” and “I intend to follow the brand’s page.” Cronbach’s alpha was 0.92.

### 3.5. Analysis

Prior to testing our main hypotheses, we calculated descriptive statistics and Pearson correlations for all key variables. We also conducted independent-sample *t*-tests to examine mean differences in these variables between age groups.

We performed hierarchical multiple regression analyses to test Hypotheses 1 through 4, which proposed effects of functional and critical consuming literacy on perceived interactivity and openness, and Research Questions 1 to 4, which proposed effects of the moderation of age group. In the first step, we conducted separate regression models of perceived interactivity and openness with the following as predictors: dummy-coded participant sex (0 = male, 1 = female, as a covariate), dummy-coded age group (0 = younger group, 1 = older group), functional consuming literacy, and critical consuming literacy. The significance of the regression coefficients for both functional and critical consuming literacy was assessed.

In the second step, interaction terms between age group and each component of consuming literacy were added to the model. We then compared the *R*^2^ values of the models to determine whether these interaction terms significantly increased the explained variance. If the interaction-included model improved model fit, we interpreted the significance of individual interaction terms. For significant interactions, we performed simple slope analyses to examine the nature of these effects between age groups.

To test Hypotheses 5 and 6, we examined the indirect effects of functional and critical consuming literacy on brand engagement, mediated by perceived interactivity and openness. These indirect effects were analyzed using the approach proposed by Hayes (2022), which calculates point estimates for direct and indirect effects based on regression coefficients. To answer Research Questions 5 and 6, we further examined the conditional indirect effects moderated by age group using the index of moderated mediation (Hayes, 2015). Statistical significance for both indirect and conditional indirect effects was determined using bootstrap confidence intervals (CIs; 20,000 iterations, bias-corrected and accelerated method). A bootstrap CI that did not include zero was considered statistically significant. For a significant index of moderated mediation, we estimated the indirect effects at different levels of age groups to illustrate the pattern of the conditional indirect effects. It is important to note that conditional indirect effects were examined only for models in which age group was found to be a significant moderator in the previous regression analyses (Research Questions 1–4). This approach offers a parsimonious alternative to structural equation modeling, especially with smaller samples. All analyses were conducted using R version 4.4.2 (R Core Team, 2024).

## 4. Results

### 4.1. Descriptive Statistics and Correlations

The descriptive statistics of and correlations between the variables are in Table 1. Analyses of group differences on key variables confirmed that the younger and older groups differed significantly. Consistently with prior research, functional consuming literacy and critical consumption literacy were lower in the younger group than in the older group: functional consuming literacy: *t*(258) = 6.68, *p* < 0.001, *d* = 0.83; critical consumption literacy, *t*(258) = 5.28, *p* < 0.001, *d* = 0.66. It was also observed that the older group evaluated the brand’s interactivity, openness, and engagement higher than the younger group: interactivity, *t*(258) = −2.12, *p* = 0.035, *d* = −0.26; openness, *t*(258) = −3.70, *p* < 0.001, *d* = −0.46; brand engagement *t*(258) = −3.89, *p* < 0.001, *d* = −0.48.

### 4.2. Multiple Regressions

We first analyzed the regression model for perceived interactivity to examine Hypotheses 1 and 2. The model without interaction terms (Table 2, Model 1) revealed that the effect of functional consuming literacy on perceived interactivity was nonsignificant, (*b* = 0.00, *SE* = 0.13, *p* = 0.995, 95% CI [−0.25, 0.25]). This indicated functional consuming literacy did not impact perceived interactivity and rejected Hypothesis 1. While the effect of critical consumption literacy was significant (*b* = 0.26, *SE* = 0.11, *p* = 0.019, 95% CI [0.04, 0.48]), the pattern was inverse against Hypothesis 2: the higher the critical consuming literacy, the higher the perceived openness. Therefore, Hypothesis 2 was not supported either.

**Figure 2:**
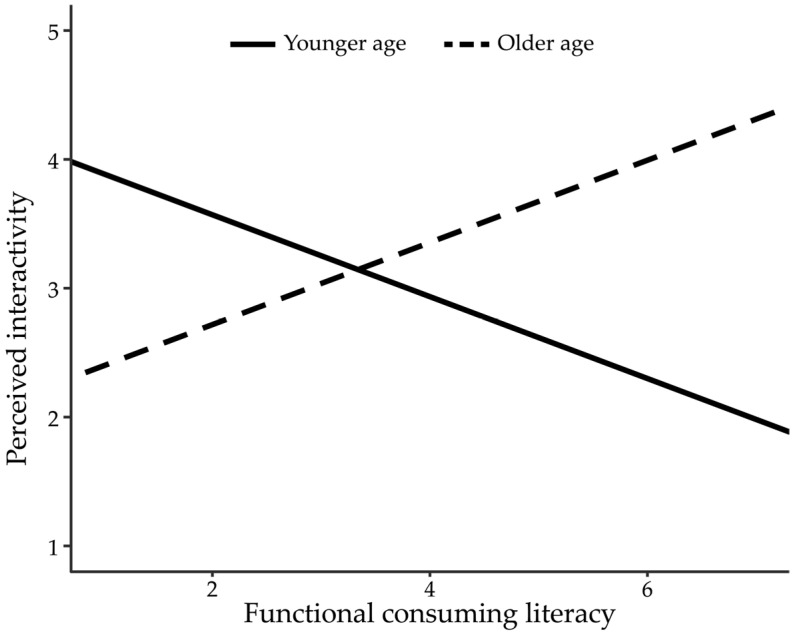
The effect of functional consumption literacy on perceived interactivity by age group.

We further examined Research Questions 1 and 2 by adding interaction terms to the regression model (Table 2, Model 2). The interaction effect between functional consuming literacy and age group was significant (*b* = 0.64, *SE* = 0.25, *p* = 0.013, 95% CI [0.13, 0.63]). This indicated that the effect of functional consuming literacy varied between the age groups. Simple slope analysis showed that the effect of functional consuming literacy was preliminarily increased perceived interactivity of the younger group (*b* = −0.32, *SE* = 0.18, *p* = 0.073, 95% CI [−067, 0.03]), but preliminarily decreased interactivity of the older group (*b* = 0.32, *SE* = 0.18, *p* = 0.083, 95% CI [−0.04, 0.68]). This pattern of interactions is visualized in Figure 2.

In the case of critical consumption literacy, the interaction term was nonsignificant (*b* = −0.18, *SE* = 0.22, *p* = 0.414, 95% CI [−0.62, 0.25]), indicating the effect of critical consuming literacy did not vary between the age groups.

To examine Hypotheses 3 and 4, the regression model for perceived openness was fitted. In the model without interaction terms (Table 3, Model 1), the effect of functional consuming literacy on perceived openness was nonsignificant (*b* = −0.18, *SE* = 0.13, *p* = 0.151, 95% CI [−0.43, −0.07]). This indicated that perceived openness was not affected by functional consuming literacy, rejecting Hypothesis 3. The effect of critical consumption literacy was significant (*b* = 0.32, *SE* = 0.11, *p* = 0.004, 95% CI [0.10, 0.53]). However, the pattern of effect was contrary to the prediction. Therefore, Hypothesis 4 was not supported.

**Figure 3:**
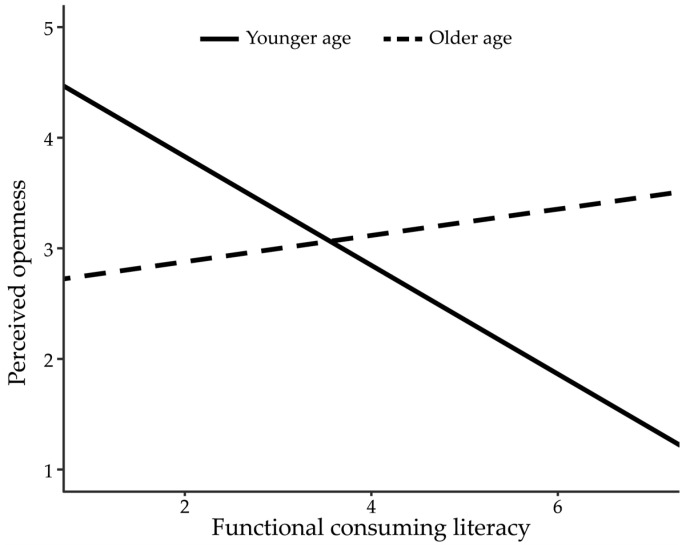
The effect of functional consumption literacy on perceived openness by age group.

The regression model with added interaction terms was further investigated to examine Research Questions 3 and 4 (Table 3, Model 2). The interaction effect between functional consuming literacy and age group was significant (*b* = 0.61, *SE* = 0.25, *b* = 0.016. 95% CI [0.11, 1.11]). This means that the effect of functional consuming literacy varied between the age groups. In the younger group, functional consuming literacy negatively impacted perceived openness (*b* = −0.49, *SE* = 0.18, *p* = 0.005, 95% CI [−0.84, −0.15]). However, the effect was nonsignificant in the older group (*b* = 0.12, *SE* = 0.18, *p* = 0.513, 95% CI [−0.24, 0.48]). This pattern of interactions is visualized in Figure 3.

The interaction term between critical consumption literacy and age group was nonsignificant (*b* = −0.12, *SE* = 0.22, *p* = 0.599, 95% CI [−0.55, 0.31]). This indicates that the effect of critical consuming literacy on perceived openness was constant between age group.

### 4.3. Indirect Effects

**Figure 4:**
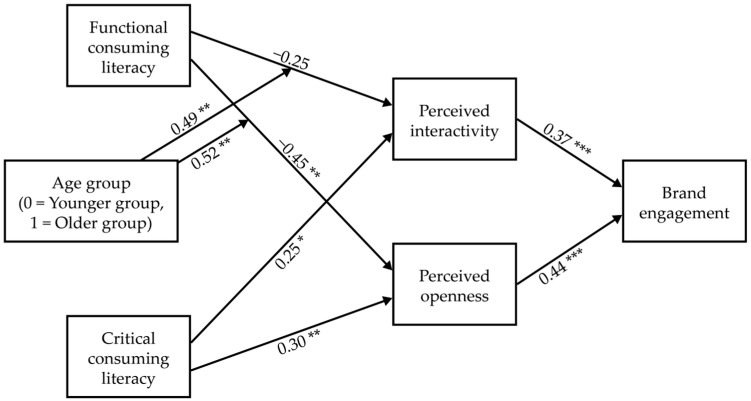
Path coefficients of the conditional indirect effect model. The coefficients in parentheses mean total effects. *** *p* < 0.001, ** *p* < 0.01, * *p* < 0.01.

We analyzed a (conditional) indirect model to examine Hypotheses 5 and 6 and Research Questions 5 and 6. Based on the results in Section 4.2, we examined the conditional indirect effect of functional consuming literacy (Research Question 5) and indirect effect of critical consuming literacy (Hypothesis 6). Each estimated path coefficient in the model is displayed in Figure 4 (detailed results of regression models can be found in Appendix B).

To examine Research Question 5, we calculated indexes of moderated mediation of age group in the indirect effects of functional consuming literacy and the 95% bootstrap CIs. The moderated mediation index through perceived interactivity was 0.18, bootstrap *SE* = 0.08, 95% CI [0.06, 0.38]. The moderated mediation index through perceived openness was 0.22, bootstrap *SE* = 0.09, 95% CI [0.08, 0.43]. As neither confidence interval included zero, it was concluded that those indirect effects significantly varied between age groups.

The pattern of conditional indirect effects is displayed in Table 4. The indirect effect of functional consuming literacy that mediated perceived interactivity was preliminarily negative in the younger group (90% CI [−0.22, −0.01]), but positive in the older group (90% CI [0.01, 0.22]). In the case of the indirect effect through perceived openness, the effect was significantly negative in the younger group, but nonsignificant in the older group. The total indirect effect was significantly negative in the younger group, but nonsignificant in the older group. Overall, functional consuming literacy significantly decreased brand engagement by mediating perceived interactivity and openness only in the younger group.

In the case of Hypothesis 6, indirect effects of critical consumption literacy that mediated both brand perceptions were statistically significant: perceived interactivity, effect = 0.09, boot *SE* = 0.05, 95% CI [0.01, 0.22]; perceived openness, effect = 0.13, boot *SE* = 0.05, 95% CI [0.04, 0.41]. The total indirect effect was also significant, effect = 0.22, boot *SE* = 0.09, 95% CI [0.04, 0.41]. This meant that critical consuming literacy, mediating perceived interactivity and openness, positively influenced brand engagement. However, the pattern was contrary to the prediction. Therefore, Hypothesis 6 was not supported.

## 5. Discussion

This study examined whether functional consuming literacy and critical consuming literacy, components of new media literacy, affect brand engagement via the mediating mechanisms of perceived interactivity and openness. Furthermore, it investigated whether these effects of new media literacy differed between various consumer age groups. The main findings of this study are as follows. First, functional consuming literacy did not exert a statistically significant direct effect on perceived interactivity or openness with a brand. However, functional consuming literacy interacted with age group to shape perceptions of both interactivity and openness. Specifically, among the younger group, functional consuming literacy negatively influenced those perceptions. In contrast, among the older group, functional consuming literacy positively affected perceived interactivity, but did not significantly impact perceived openness.

These findings can be understood through the socioemotional selectivity theory (Carstensen, 2006). As highlighted in the theoretical background, individuals with a longer perceived remaining lifespan are more likely to prioritize acquiring new information, leading to a greater focus on negative information. Conversely, individuals with a shorter perceived lifespan prioritize regulating their emotions to enhance psychological well-being, resulting in stronger attention to positive information. In other words, socioemotional goals that direct the allocation of cognitive resources vary depending on individuals’ perceptions of their remaining lifespan (Carstensen & DeLiema, 2018). Functional consuming literacy refers to the ability to acquire and comprehend information through new media and encompasses the cognitive resources required for such tasks (Castañeda et al., 2020). Considering socioemotional selectivity theory, functional consuming literacy in younger groups is used for biased processing of negative aspects of stimuli (e.g., a hotel brand’s social media posts), whereas in older groups, it may facilitate the processing of positive aspects of the same stimuli. Consequently, the younger group evaluated the brand’s interactivity and openness more negatively, while the older group exhibited more favorable evaluations.

Second, critical consuming literacy was identified as a positive determinant of perceived interactivity and openness between the age groups. This finding suggests that consumers with high critical consuming literacy may assess a brand’s interactivity and openness using the brand’s observable behaviors, as well as their normative understanding of interactions within new media environments. Unlike offline interactions, interactions within new media environments often occur asynchronously: users do not need to respond to their counterparts immediately. Such characteristics of new media make non-responsiveness in user interactions plausible (Nardi et al., 2000). Moreover, according to media richness theory, online communication tends to restrict the breadth and depth of self-disclosure compared to face-to-face communication due to its limitations in effectively conveying factual information (Hwang & Lee, 2009; Nguyen et al., 2012). As a result, interactions in new media environments generally exhibit lower levels of interactivity and openness than those in offline settings. In this context, consumers with high critical consuming literacy interpret messages within the broader contexts. Consequently, they may have relied on normative knowledge of new media interactions when evaluating a brand’s interactivity and openness, potentially applying a more lenient standard.

Third, the mediating roles of perceived interactivity and openness in the relationship between functional consuming literacy and brand engagement differed between the age groups. Among the younger group, functional consuming literacy exerted a negative indirect effect on brand engagement through both perceived interactivity and openness. In contrast, for the older group, functional consuming literacy positively impacted brand engagement via perceived interactivity alone. In comparison, critical consuming literacy positively impacted brand engagement in both age groups, with perceived interactivity and openness as mediators. These findings align with prior research identifying perceived interactivity and openness as critical antecedents of brand engagement (Labrecque, 2014). Furthermore, the results underscore that both functional consuming literacy and critical consuming literacy extend their influence beyond brand perceptions to shape consumer behaviors toward brands.

Lastly, the findings of this study suggest that functional consumption literacy should be considered separately from established persuasive knowledge. In general, the activation of persuasive knowledge varies based on individual differences, such as cognitive resources and age (Hardesty et al., 2007). Research has shown that persuasive knowledge matures as cognitive resources increase and with advancing age. However, because the activation of persuasive knowledge is domain-specific, even when general persuasive knowledge is activated, the specific context of the domain must still be considered. According to this study, persuasive knowledge related to advertising may have differing effects depending on pricing strategies or direct sales tactics (Rahmani, 2023, p. 15). Consequently, the results suggest that even when individuals possess high functional consumption literacy, it reflects their ability to utilize new media rather than their knowledge of sales strategies. Therefore, the study implies that individuals with high functional consumption literacy may not resist brand activities on social media, but rather engage with them more proactively. These findings indicate that while new media literacy and persuasive knowledge may appear similar as cognitive resources, they should be regarded as distinct types of cognitive resources.

### 5.1. Practical Implications

The findings of this study have significant practical implications for the development of differentiated brand communication strategies that consider consumers’ functional and critical consuming literacy as well as their age. Specifically, younger consumers may interpret new media messages in ways that negatively influence their perceptions of brand interactivity and openness, thereby leading to lower levels of brand engagement. To address the adverse effects of functional consuming literacy among younger consumers, brand messages should incorporate explicit cues designed to enhance perceptions of interactivity and openness. For instance, implementing strategies such as personalized messaging and tailored advertisements can signal sustained engagement with consumers, thereby fostering more favorable perceptions among younger consumers. On the other hand, for older consumers, higher functional consuming literacy tends to result in stronger perceptions of brand interactivity. Therefore, brands should actively promote social media communications tailored specifically to older consumers who demonstrate familiarity with new media devices. For example, brands could prioritize and optimize their presence on social media platforms that cater to older groups, engaging in strategically curated brand activities that align with their preferences.

In addition, “design thinking” has garnered significant attention as an effective strategy for advertising in the new media environment (Brown, 2008). This approach positions consumers as active collaborators in the development of advertising strategies, with the aim of gaining a deeper understanding of their needs. To implement effective advertising strategies in the new media context, a high level of literacy among consumers is essential. The findings of this study suggest that critical consuming literacy, rather than functional consuming literacy, is more likely to lead to favorable perceptions of brand activities and higher levels of brand engagement. Therefore, it is expected that advertising strategies can be more effective when collaborating with consumers who possess high levels of critical consuming literacy in the new media environment. Additionally, with regard to functional consuming literacy, consumer perceptions of brand activities differ depending on their age group, making it important to reference the results of this study when selecting target consumers for collaboration. Consequently, identifying the key elements to emphasize in advertising based on these differences becomes crucial. Ultimately, this study provides practical implications by demonstrating how a nuanced understanding of the interaction between literacy types and age among target consumers can inform the development of more sophisticated and effective advertising strategies.

Lastly, the study confirmed that critical consuming literacy positively influences perceived interactivity, openness, and brand engagement across age groups. This implies that encouraging consumers’ critical thinking through carefully crafted brand messages or reinforcing normative communication practices on social media can effectively elicit positive consumer responses. Unlike traditional media, social media provides the opportunity to convey two-sided messages about a brand or product rather than one-sided ones, thereby stimulating consumers’ critical thinking. Such an approach may mitigate the activation of persuasion knowledge while simultaneously enhancing consumer trust.

### 5.2. Limitations and Suggestions

The limitations of this study are as follows. First, the study observed participants’ responses to a fictional brand to control their prior knowledge or experiences with brands. Moreover, because a brand’s social media behavior can influence perceptions of interactivity and openness, neutral brand posts were selected based on a preliminary survey to ensure a balanced evaluation across both variables. While this procedure enhanced the internal validity of the study’s findings, it may not fully reflect the way consumers would typically engage with real-world brand posts on social media. Therefore, to improve the external validity of the results, future studies should incorporate real-world brands and social media content that demonstrate varying degrees of interactivity and openness.

Second, this study did not examine prosuming literacy, which relates to users’ direct participation in new media. This is because the study focused on perceived interactivity and openness, which are more closely related to the processing of brand messages, rather than users’ direct media participation. However, prosuming literacy is more relevant when research examines effortful engagement action. For example, consumers highly engaged with a brand may participate in short-form challenges and contests or engage in collective actions related to the brand. Thus, future research should carefully select the dimensions of new media literacy that align with the type of consumer response or behavior being studied.

Lastly, this study did not account for individual differences beyond age and gender. Future research should address potential individual difference moderating factors, such as self-construal, which may influence how new media literacy shapes perceptions of interactivity and openness. For instance, E.-H. Kim et al. (2019) suggest that consumers’ self-construal—whether independent or interdependent—may lead to differences in their perceptions of these dimensions. Additionally, self-construal can vary by age (Barak et al., 2011; Yeung et al., 2008) and across cultures (Barak et al., 2011; E.-H. Kim et al., 2019), further impacting these perceptions. Exploring these moderating effects in future studies would provide a deeper understanding of how new media literacy influences brand engagement.

## 6. Conclusions

This study highlights the significant impact of both functional and critical consuming literacy on brand engagement, a key performance indicator of brand communication in the new media era. The findings suggest that new media literacy should be considered a crucial variable in understanding and predicting consumer behavior in the context of new media, emphasizing its relevance beyond traditional frameworks. Additionally, this research reveals that the effect of functional consuming literacy may vary across different age groups. This insight challenges the conventional view of age as merely a predictive factor for new media literacy and contributes to a more nuanced understanding of the relationship between age and new media literacy.

## Figures and Tables

**Table 1:** Descriptive statistics of and correlations between the variables.

Variables		*M* (*SD*)		1	2	3	4	5
		Younger Group	Older Group					
1	FCL	6.05 (0.89)	5.28 (0.96)	-	0.76	0.36	0.27	0.18
2	CCL	5.64 (1.03)	4.94 (1.10)	0.67	-	0.33	0.31	0.19
3	Perceived interactivity	4.22 (1.48)	4.58 (1.21)	−0.03	0.11	-	0.73	0.65
4	Perceived openness	3.85 (1.50)	4.47 (1.14)	−0.13	0.05	0.78	-	0.70
5	Brand engagement	3.48 (1.60)	4.17 (1.34)	−0.02	0.02	0.64	0.65	-

Note. FCL = functional consuming literacy, CCL = critical consumption literacy. The upper and lower triangular matrices in the correlation table indicate correlations for the younger and the older groups, respectively. All the correlations |*r*| > 0.13 are significant at *p* < 0.05.

**Table 2:** The regression analysis of perceived interactivity.

Variables	Model 1				Model 2			
	*b*	*SE*	*p*	95% CI	*b*	*SE*	*p*	95% CI
Constant	2.56	0.59	<0.001	[1.58, 3.78]	4.20	0.81	<0.001	[2.42, 5.69]
Sex	0.12	0.17	0.479	[−0.21, 0.44]	0.15	0.16	0.365	[−0.17, 0.47]
Age group	0.54	0.18	0.003	[0.19, 0.89]	−2.13	1.04	0.042	[−4.17, −0.08]
FCL	0.00	0.13	0.995	[−0.25, 0.25]	−0.32	0.18	0.073	[−0.67, 0.03]
CCL	0.26	0.11	0.019	[0.04, 0.48]	0.33	0.15	0.029	[0.03, 0.63]
Age group × FCL					0.64	0.25	0.013	[0.13, 1.14]
Age group × CCL					−0.18	0.22	0.414	[−0.62, 0.25]
	*R*^2^ = 0.06, *F*(4, 255) = 4.27, *p* = 0.002				Δ*R*^2^ = 0.03, *F*(2, 253) = 4.08, *p* = 0.018			

Note. FCL = functional consuming literacy, CCL = critical consumption literacy, CI = confidence interval.

**Table 3:** The regression analysis of perceived openness.

Variables	Model 1				Model 2			
	*b*	*SE*	*p*	95% CI	*b*	*SE*	*p*	95% CI
Constant	3.17	0.55	<0.001	[2.07, 4.28]	4.81	0.80	<0.001	[3.23, 6.39]
Sex	0.02	0.16	0.898	[−0.30, 0.35]	0.05	0.16	0.756	[−0.27, 0.37]
Age group	0.69	0.18	<0.001	[0.34, 1.04]	−2.17	1.03	0.036	[−4.20, −0.15]
FCL	−0.18	0.13	0.151	[−0.43, 0.07]	−0.49	0.17	0.005	[−0.84, −0.15]
CCL	0.32	0.11	0.004	[0.10, 0.53]	0.35	0.15	0.019	[0.06, 0.65]
Age group × FCL					0.61	0.25	0.016	[0.11, 1.11]
Age group × CCL					−0.12	0.22	0.599	[−0.55, 0.31]
	*R*^2^ = 0.083, *F*(4, 255) = 5.78, *p* < 0.001				Δ*R*^2^ = 0.03, *F*(2, 253) = 4.42, *p* = 0.013			

Note. FCL = functional consuming literacy, CCL = critical consumption literacy, CI = confidence interval.

**Table 4:** The conditional indirect effects of functional consuming literacy.

Mediator	Moderator	Effect	Boot *SE*	95% CI
Perceived interactivity	Younger group	−0.09	0.06	[−0.24, 0.00]
	Older group	0.09	0.06	[−0.01, 0.25]
Perceived openness	Younger group	−0.19	0.09	[−0.39, −0.05]
	Older group	0.02	0.06	[−0.08, 0.15]
Total indirect effect	Younger group	−0.28	0.12	[−0.54, −0.05]
	Older group	0.12	0.10	[−0.08, 0.32]

Note. CI = confidence interval.

**Figure A1:**
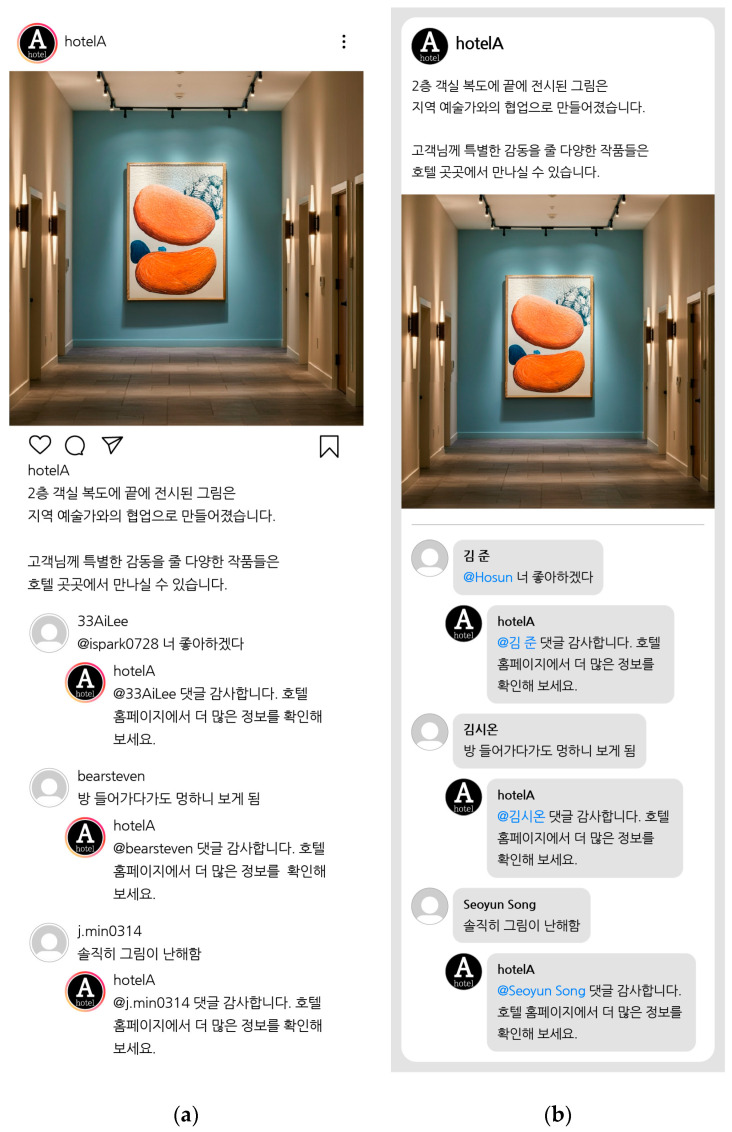
The hypothetical hotel brand’s (**a**) Instagram and (**b**) Facebook posts. The stimuli are identical except for the template and users’ nicknames. The main text is as follows: “The artwork at the end of the 2nd floor aisle was created in cooperation with a local artist. You can find inspiring artworks all over the hotel.” The three comments are as follows: “You might like this”, “I could stare at it for hours”, and “TBH, I don’t really get this painting”. The brand gives the same comments to each: “Thanks for your comment. Get more information on the hotel’s homepage.”.

## Data Availability

The raw data supporting the conclusions of this article will be made available by the authors upon request.

## Appendix B

**Table A1:** The results of regression analysis for conditional indirect effect model.

Outcomes	Predictors	*b*	*SE*	*p*	95% CI	
Brand engagement	Constant	5.69	0.87	<0.001	[3.97, 7.41]	*R*^2^ = 0.13, *F*(5, 254) = 7.40, *p* < 0.001
	Sex	−0.18	0.18	0.310	[−0.53, 0.17]	
	Age group	−2.91	1.12	0.010	[−5.11, −0.71]	
	FCL	−0.66	0.17	<0.001	[−0.99, −0.32]	
	CCL	−0.33	0.12	0.006	[0.09, 0.56]	
	Age group × FCL	−0.63	0.19	0.001	[0.25, 1.01]	
Perceived interactivity	Constant	4.28	0.81	<0.001	[2.70, 5.87]	*R*^2^ = 0.08, *F*(5, 254) = 5.00, *p* < 0.001
	Sex	0.14	0.16	0.394	[−0.18, 0.46]	
	Age group	−2.24	1.03	0.031	[−4.26, −0.21]	
	FCL	−0.25	0.16	0.110	[−0.56, 0.06]	
	CCL	0.25	0.11	0.026	[0.03, 0.46]	
	Age group × FCL	0.49	0.18	0.007	[0.14, 0.84]	
Perceived openness	Constant	4.86	0.80	<0.001	[3.29, 6.43]	*R*^2^ = 0.11, *F*(5, 254) = 6.48, *p* < 0.001
	Sex	0.04	0.16	0.782	[−0.27, 0.36]	
	Age group	−2.24	1.02	0.028	[−4.25, −0.24]	
	FCL	−0.45	0.15	0.004	[−0.75, −0.14]	
	CCL	0.30	0.11	0.006	[0.08, 0.51]	
	Age group × FCL	0.52	0.18	0.004	[0.17, 0.86]	
Brand engagement	Constant	1.97	0.68	0.004	[0.63, 3.32]	*R*^2^ = 0.54, *F*(7, 252) = 42.88, *p* < 0.001
	Sex	−0.25	0.13	0.052	[−0.51, 0.00]	
	Age group	−1.10	0.82	0.180	[−2.72, 0.51]	
	FCL	−0.37	0.13	0.004	[−0.62, −0.12]	
	CCL	0.11	0.09	0.222	[−0.07, 0.28]	
	Interactivity	0.37	0.07	<0.001	[0.23, 0.52]	
	Openness	0.44	0.07	<0.001	[0.29, 0.58]	
	Age group × FCL	0.23	0.14	0.111	[−0.05, 0.51]	

Note. FCL = functional consuming literacy, CCL = critical consumption literacy, CI = confidence interval.
